# Development of a workplace intervention for sick-listed employees with stress-related mental disorders: Intervention Mapping as a useful tool

**DOI:** 10.1186/1472-6963-7-127

**Published:** 2007-08-15

**Authors:** Sandra H van Oostrom, Johannes R Anema, Berend Terluin, Anita Venema, Henrica CW de Vet, Willem van Mechelen

**Affiliations:** 1 EMGO Institute, VU University Medical Center, Amsterdam, The Netherlands; 2 Department of Public and Occupational Health, VU University Medical Center, Amsterdam, The Netherlands; 3 Body@Work, Research Center Physical Activity, Work and Health, TNO-VU, Amsterdam, The Netherlands; 4 Research Center for Insurance Medicine AMC-UWV-VU University Medical Center, Amsterdam, The Netherlands; 5 Department of General Practice, VU University Medical Center, Amsterdam, The Netherlands; 6 TNO Quality of Life, Hoofddorp, The Netherlands

## Abstract

**Background:**

To date, mental health problems and mental workload have been increasingly related to long-term sick leave and disability. However, there is, as yet, no structured protocol available for the identification and application of an intervention for stress-related mental health problems at the workplace. This paper describes the structured development, implementation and planning for the evaluation of a return-to-work intervention for sick-listed employees with stress-related mental disorders (SMDs). The intervention is based on an existing successful return-to-work intervention for sick-listed employees with low back pain.

**Methods:**

The principles of Intervention Mapping were applied to combine theory and evidence in the development, implementation and planning for the evaluation of a participatory workplace intervention, aimed at an early return-to-work for sick-listed employees with SMDs. All stakeholders were involved in focus group interviews: i.e. employees recently sick-listed with SMDs, supervisors and occupational health professionals.

**Results:**

The development of the participatory workplace intervention according to the Intervention Mapping principles resulted in a structured return-to-work intervention, specifically tailored to the needs of sick-listed employees with SMDs. Return-to-work was proposed as a behavioural change, and the Attitude – Social influence – self-Efficacy model was identified as a theoretical framework. Stakeholder involvement in focus group interviews served to enhance the implementation. The cost-effectiveness of the intervention will be evaluated in a randomised controlled trial.

**Conclusion:**

Intervention Mapping was found to be a promising method to develop interventions tailored to a specific target group in the field of occupational health.

**Trial registration:**

ISRCTN92307123

## Background

To date, mental health problems have been increasingly related to long-term sick leave and disability [1], but work-related mental health problems and workload are not easily discussed in the workplace [2]. Even when the afflicted employees are in contact with health care providers they find these problems difficult to mention and discuss [3]. The results of some recent studies indicate that sick leave due to this type of complaints can be reduced by activating interventions: an activating intervention supervised by the occupational physician (OP) [4], a combined individual and workplace intervention supervised by labour experts [5], occupational therapy for major depression [6], and a minimal intervention strategy, concentrating on work resumption, provided by the general practitioner [7]. These studies showed that earlier return-to-work (RTW) is not associated with an increase or decrease in complaints [4-7], but is seen as part of the recovery process. It could assist an employee to regain control and to recover more quickly. However, there is still no structured protocol available to identify (work-related) mental health problems, to discuss them, and to find solutions to facilitate RTW.

A protocol to facilitate RTW is available for employees with low back pain, and a recent study reported promising results [8-10]. This Participatory Workplace (PW) intervention is based on principles used in Participatory Ergonomics [11]. The PW intervention consists of a stepwise process to identify and solve barriers for RTW, based on consensus between the sick-listed employee and his/her supervisor about a plan to facilitate RTW. Employees are first referred to an RTW coordinator (in most cases an ergonomist) by their OP. Then, the employee and supervisor identify barriers for RTW separately in structured conversations with the RTW coordinator, based on a task-analysis. In a third conversation the employee, the supervisor and the RTW coordinator brainstorm together to find solutions, resulting in a plan for RTW, based on consensus. One of the essential features of the protocol is that the RTW coordinator's role is predominantly that of guiding the process, not that of an occupational health (OH) professional who decides what is wrong and what should be done about it. The actions planned are those that both the employee and the supervisor have proposed and decided upon.

This protocol accelerated RTW by 27 days [9,12] and both the compliance and satisfaction with the intervention were good for employees and OH professionals [10]. This PW intervention may also be applicable for employees who are on sick leave due to mental workload and stress [13]. Secondary analysis of participants with both low back pain and problems related to mental workload or stress, showed that the intervention made it possible to identify mental workload and stress issues, to discuss them and to cope with them. Obstacles for RTW related to mental health were identified as job strain, work atmosphere and personality characteristics of the worker. Compared with obstacles related to physical workload, the solutions for mental workload and stress were more often found in job content and work organisation. Based on their results, Jettinghoff recommended that a prospective study should be carried out to assess the applicability and effectiveness of the PW intervention for sick-listed employees with stress-related mental disorders (SMDs) [13].

It was decided to develop a workplace intervention for sick-listed employees with SMDs, based on the promising RTW intervention for low back pain. However, the implementation of evidence-based RTW interventions in occupational health has been difficult [14], due to the absence of key stakeholder involvement in the development of such interventions [15-18]. Goldenhar et al. suggest the development of a research agenda to carry an OH intervention through all three phases: development, implementation and evaluation [14]. In health education and health promotion research, interventions have been developed and implemented in a very structured manner. A structured process such as Intervention Mapping (IM) is often applied in the development of an intervention in this field of research. IM includes both knowledge obtained from the literature and involvement of key stakeholders to develop, implement and evaluate an intervention [19,20]. The application of IM in the development of interventions in OH research is a challenge, and to our knowledge this will be the first time such a process was used to develop an RTW intervention in the field of OH.

This paper describes the process of adjusting the protocol of the PW intervention for sick-listed employees with SMDs, and applying IM principles that make it possible to tailor the intervention to this specific target group.

## Methods

**Figure 1:**
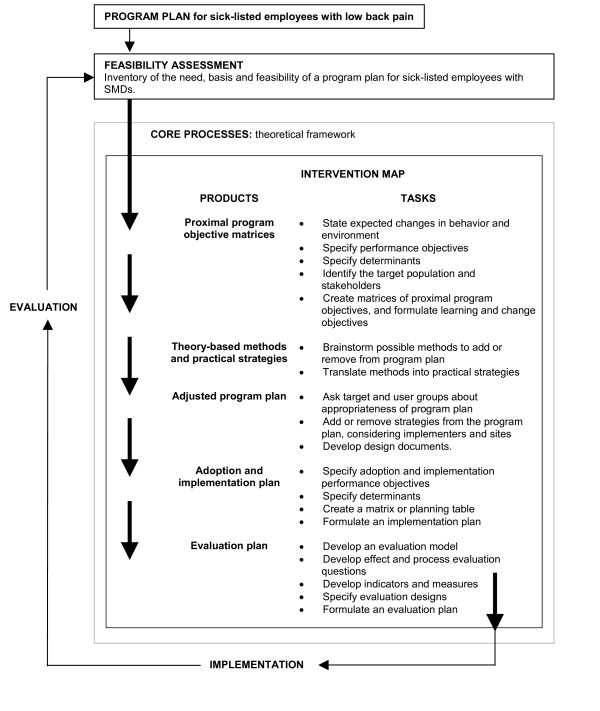
**Modified Intervention Mapping process**. Modified Intervention Mapping process aimed at adjusting the Participatory Workplace intervention for employees with stress-related mental disorders (based on the Intervention Mapping process described by Bartholomew et al. [20]).

IM is a stepwise approach in the development of interventions based on a combination of theory and evidence [20]. IM is not a theoretical or conceptual framework, but rather a description of a logical planning process. It consists of the five steps presented in Figure 1. In our study, we already had a draft of a program plan, e.g. the PW protocol for low back pain. Therefore, figure 1 is a modified version of the original IM process [20]. IM is not rigid; it is an iterative process which makes it possible to go back to earlier steps or forward if necessary through new perspectives. Collaboration between the developers and the users of the intervention and the people for whom the program will be designed is a basic assumption. IM originated in primary care prevention, whereas this study focuses on the prevention of work disability.

### Feasibility assessment

Before starting the IM process, a feasibility assessment was carried out in the three participating large companies: a university, a hospital and a steel company. A multidisciplinary project group (three OPs, a psychologist, a general practitioner and an epidemiologist) was responsible for making an inventory of the need and basis in companies for a PW intervention for sick-listed employees with SMDs. In formal meetings attended by the project group and stakeholders the need for an RTW intervention was discussed: two meetings with a group of human resource managers and personnel officials, two meetings with a group of OH professionals, and two meetings with the works council. The protocol has already been found to be feasible for low back pain. The feasibility of the protocol for SMDs was also addressed in the focus group interviews. In addition to the inventory that was made of the need, support system and feasibility of a PW intervention, information derived from these meetings was also used to assess co-operation in IM and implementation of the protocol in the companies.

### Core processes: theoretical framework

The core process of IM is intended to access theory and evidence in intervention planning. The literature was non-systematically reviewed to select an appropriate theoretical framework for the program plan. This framework with accompanying determinants was applied throughout the various steps of IM.

### Step 1 – Define specific intervention objectives

In step 1 of the IM process an overall program objective for the intervention was formulated, and the target group and stakeholders were specified. Identification of the primary stakeholders is a critical step in designing interventions in OH [21,22]. The performance objectives that were specified were based on the program objectives. Performance objectives are the effects of the intervention on the target population in terms of things which should be learned or specific behavior which should be changed. The matrices that combine performance objectives with determinants of RTW were then developed to enable translation to specific intervention goals: i.e. learning and change objectives. Learning objectives answer the question: what does the target population need to learn or acquire with regard to a specific determinant to achieve the performance objective? Change objectives answer the question: what needs to be changed (in the environment) for the target population to achieve the performance objective?

### Step 2 – Select suitable theoretical methods and practical strategies

In step 2 of the IM process, a review of the literature and a brainstorm session in the multidisciplinary project group resulted in the selection of appropriate methods and practical strategies to address each learning or change objective. Theoretical methods are general techniques or processes, derived from empirical evidence that describe the association between an intervention and a change in behavioural determinants. Practical strategies are defined as techniques for the application of the theoretical methods. This results in a matrix in which theoretical methods are matched with practical strategies for each determinant. As a PW intervention for low back pain already exists, this was done by adapting the original intervention protocol to the new target group.

### Step 3 – Design a program plan

Step 3 of the IM process is intended to formulate a program plan. Before a program plan could be formulated program users e.g. recently sick-listed employees with SMDs, the supervisors and the OH professionals were invited in three separate focus group interviews to express their views on the preconditions for applying the PW intervention for sick-listed employees with SMDs. OPs from the participating companies were asked to select and recruit employees and accompanying supervisors who met the requirements of our definition of SMDs.

The focus group interviews with 8 to 12 participants lasted for 90 minutes. Each focus group discussion was tape-recorded and transcribed. Ethical approval for the focus group interviews was obtained from the Medical Ethics Committee of the VU University Medical Center. Participants signed a privacy agreement to declare the following: voluntary participation, no transmittal of information to others, and permission for processing the information for the development of the protocol.

At the start of each focus group the PW intervention for sick-listed employees with low back pain was presented and the theoretical framework was introduced (SHO). Specific statements (formulated after consultation with the multidisciplinary project group) were then presented and initiated through the group moderator (JRA/WM), after which all participants were invited to express their views. The group moderator was responsible for summarizing and verifying what was said in the discussion about each statement.

Statements were formulated about the following issues:

• Equality, safety and support in discussions about RTW.

• The role of the RTW coordinator.

• Preconditions needed for an employee to participate in the PW intervention.

• Expected barriers for implementing the PW intervention for sick-listed employees with SMDs.

Guided by the matrices developed in steps 1 and 2 and the results of the focus group interviews, the multidisciplinary project group selected and integrated components for a PW program for this target group.

### Steps 4 and 5 – Design an implementation and evaluation plan

In step 4 of the IM process, a plan for the implementation and adoption of the program was designed, including implementation objectives, methods and strategies. Finally, in step 5, an evaluation plan and the corresponding evaluation measures were identified and developed.

## Results

### Feasibility assessment

The need and basis in companies for a PW intervention was commonly shared between human resource managers, personnel officials, OH professionals and the works council. Discussions about mental health problems in the workplace are frequently avoided by employees and supervisors since there is no structured protocol available for them to discuss the mental strain at work. Therefore, at present, there is no uniformity in applying work adaptations. The strength of the protocol is thought to be that it provides an opportunity for facilitating a structured conversation between the supervisor and the sick-listed employee, guided by an intermediary, in an early stage of sick leave. This is in line with general Dutch guidelines that recommend maintaining regular contacts between employee and employer after reporting sick, early and adequate diagnosis and intervention by health care providers, and early activation at home and at work [1]. The PW intervention concurs with recent changes in the Dutch law, and also the political and societal attention that is being paid to the prevention of sick leave due to mental health problems. In spite of the time needed to apply the protocol, and the accompanying costs for employers, the PW intervention is considered to be appropriate and necessary for employees with SMDs.

### Core processes: theoretical framework

Several studies have indicated that employees can RTW despite symptoms, and that work resumption does not necessarily increase the symptoms [4,5,12,13]. However, most RTW interventions focus on the treatment of medical conditions, expecting this to facilitate RTW. Moreover, predictors for long-term absenteeism, being of a psychosocial nature, are multifactorial [23]. Therefore, a focus on RTW behavior rather than on the medical condition of a worker is important in the development of interventions [22,24-26].

**Figure 2:**
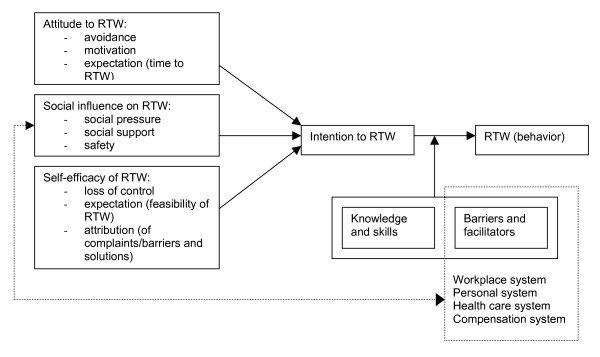
ASE model applied to RTW.

According to the operant conditioning theory [27], overt behavior that accompanies pain (e.g. complaining, use of medical services, sick leave) can be conditioned. In the field of health promotion research, behavioral change models are frequently used in the development and implementation of health promotion interventions. An example of a determinant model that has been applied to various types of health-related behavior is the Attitude-Social influence-self-Efficacy (ASE) model [28,29]. This model is derived from the Theory of Planned Behavior [30], and contains three categories of determinants of behavior: attitude, social influence and self-efficacy (Figure 2). Applying this model to RTW behavior, its concepts have the following meaning: attitude towards RTW is what an individual thinks and expresses about RTW for him or herself; social influence is what other people think about RTW for this individual; an individual's confidence of successful RTW defines the concept of self-efficacy. Recent literature shows that positive attitudes to RTW and highly motivated employees are likely to be essential determinants for success [3,31-33]. Social influence could be accomplished by a social network which supports RTW and guarantees safety and equality in the process [32,34,35]. Support from supervisors is especially predictive for both short and long periods of absence [31,36]. Recovery expectations are predictive of the duration of sickness absence [37-39]. Nieuwenhuijsen et al. suggested that positive recovery expectations could represent the self-efficacy expectations of employees [39]. In addition, several authors emphasise the role of self-efficacy in the RTW process, and suggest that it needs to be investigated [32,39-41]. Attitude, social influence and self-efficacy determine the intention with regard to RTW in the ASE model. RTW behavior is not determined by intention only, but also depends on barriers and facilitators and on the knowledge and skills needed to achieve RTW. Obviously, the intervention is aimed to change the determinants for intention into behavior and to remove these barriers for RTW. The step from intention to actual RTW behavior is assumed to be very essential in the prevention of work disability, since all the systems involved within a societal context (workplace system, health care system, personal system and compensation system [42,43]) can also influence the achievement of RTW in a supportive or obstructive way.

### Step 1 – Define specific intervention objectives

#### Program objective

Information collected in the needs assessment resulted in the formulation of a main objective for this intervention program: reduction of long-term sick leave and disability for sick-listed employees with SMDs. Employees with SMDs should achieve RTW early and safely by reducing barriers for RTW that are work-related. The resulting work adaptations could be directed to workplace, work organisation, working conditions, working relationships or work environment (mental and/or physical workload).

#### Target group and stakeholders

The intervention target on sick-listed employees with SMDs and their direct supervisors. This is in accordance with the Dutch guidelines, which promote an early RTW for employees with SMDs [1,44]. SMDs are defined by elevated levels of distress and psychosocial disfunctioning. Distress results from the effortpeople have to put into coping with stressors in order to maintain their habitual level of psychosocial functioning [45,46]. Severe distress, however, may lead to a breakdown in coping, resulting in psychosocial disfunctioning (i.e. sick leave). The combination of distress and psychosocial disfunctioning is denoted demoralisation [47,48], or in more popular language "nervous breakdown" [49,50]. Contrary to a categorical diagnosis of "adjustment disorder", which should not be made for specific anxiety or mood disorders [51], demoralisation does not stand for a diagnostic category, but it refers to an important dimension of mental disorders that cuts across all established diagnostic categories [46,52]. Employees with severe psychiatric disorders (e.g. mania or psychosis) were excluded from the IM process and subsequently also from the intervention.

With regard to the goal of an RTW intervention, the most important stakeholders are employees, employers and health care providers (in this case OH professionals). Labour representatives (for example unions) and insurers [25] are also important, but not essential for the PW intervention, since they are not involved in workplace interventions for individual employees in the Netherlands. Involvement of all the stakeholders in the development of an RTW protocol is important because each stakeholder operates within a set of economic, social and/or legislative contexts [16,25]. Therefore each group of stakeholders was invited to participate in a separate focus group interview; i.e. recently sick-listed employees with SMDs, supervisors and OH professionals.

#### Performance objectives

The performance objectives selected by the employees are presented in Table 1. The six performance objectives were formulated based on the structure of the PW intervention for low back pain.

**Table 1:** Performance objectives

Performance objectives for the reduction of long-term sick leave and disability for sick-listed employees with SMDs.
1. Employee learns the consequences of long-term sick leave and disability due to SMDs.
2. Employee is able to identify and prioritise (mental and physical workload) barriers for a safe and early RTW.
3. Employee is able to discuss barriers for a safe and early RTW with the supervisor and the RTW coordinator.
4. Employee is able to identify and prioritise solutions for barriers for a safe and early RTW.
5. Employee is able to discuss (mental and physical workload) solutions for an early RTW with the supervisor and the RTW coordinator.
6. Employee discusses the implementation plan with the supervisor and the RTW coordinator.

Performance objectives for the reduction of long-term sick leave and disability for employees with SMDs.

#### Determinants of performance objectives

Important and changeable determinants of these performance objectives were selected, based on the literature described in the core processes. The ASE model was used to describe factors influencing a change in RTW behavior. Other important determinants are knowledge of disability policies, recognition of the risk of long term disability, and the necessary skills to discuss issues related to RTW.

#### Learning and change objectives

A matrix with learning and change objectives was created, based on evidence from the literature and the perspectives of the different stakeholders. Table 2 presents an example of learning objectives pertaining to the performance objective: the employee is able to identify and prioritise solutions for barriers for a safe and early RTW. Table 3 presents an example of change objectives pertaining to the performance objective: the employee is able to identify and prioritise (mental and physical workload) barriers for a safe and early RTW.

**Table 2:** Example of learning objectives

**Performance objectives for the employee**	**Learning objectives**		
	
	**Risk perception and knowledge**	**Self-efficacy**	**Skills**
Employee is able to identify and prioritise solutions for bariers for a safe and early RTW.	Employee learns about possible solutions for RTW.	Employee offers solutions for RTW.	Employee thinks in broad outline and free associative for solutions. Employee explains solutions to the supervisor and the RTW coordinator. Employee respects solutions for RTW explained by the supervisor and the RTW coordinator.

Learning objectives based on the combination of a performance objective and determinants.

**Table 3:** Example of change objectives

**Performance objectives for the employee**	**Change objectives**	
	
	**Safety and equality**	**Support**
Employee is able to identify and prioritise (mental and physical workload) barriers for a safe and early RTW.	RTW coordinator explains how to identify and prioritise barriers.	OP provides tools to identify stressors.

Change objectives based on the combination of a performance objective and determinants.

### Step 2 – Select suitable theoretical methods and practical strategies

A review of the literature produced some useful theoretical methods which can influence the identified determinants: transfer of information [53] (for knowledge), verbal persuasion [54] (for attitude), and an intermediary person [55] (for safety and equality). Additionally, during a brainstorm session the multidisciplinary project group selected other useful methods: active processing of information (for knowledge and risk perception), guided practise (for skills), positive reinforcement (for self-efficacy), check for comprehension (for outcome expectations), create openness and respect (for safety and equality), prevent inhibition and encourage support from work environment (for support). Some strategies are already included in the protocol, but are of great importance, and are therefore also presented in Table 4.

**Table 4:** Theoretical methods and practical strategies

**Determinant**	**Methods from theory**	**Strategy**	**Tools/Materials**
Risk perception and knowledge	Passive learning/providing information	Providing written and verbal information	Letter sent to E explaining the research
			OP explains that early RTW does not increase complaints
			OP explains about risk of ending in work disability scheme
			Researcher explains PW intervention in phone call and sends invitation with brochure, OP also explains and RC guides PW intervention
			RC contributes to brainstorm for solutions
	Active processing of information	Evaluating understanding	OP instructs inventory of stressors to E as home assignment.
			Inventory of barriers for RTW in PW intervention.
Attitude	Verbal persuasion	Providing arguments	OP provides arguments why sick leave does not have to be experienced passively, change in behavior
			OP and RC emphasise the importance of consensus
Skills	Guided practise	Guided practise	Practise explanation of barriers to S with RC.
Self-efficacy	Positive reinforcement	Providing feedback	RC focuses on work abilities of E
		Evaluation	RC evaluates by phone
Outcome expectations	Check for comprehension	Ask E to summarize	OP and RC ask E to summarize expectations with regard to PW intervention
Safety and equality	Guidance by independent person	Train RC to guide PW intervention	Train RC to guide PW intervention
	Create openness and respect	Respect each others barriers and solutions	RC avoids discussion between E and S about truth of barriers
Support	Prevent inhibition	Avoid fixed appointments about RTW before starting PW intervention	OP does not establish RTW date before starting PW intervention
	Support from work environment	Participation of supervisor in RTW process	Increased participation of supervisor in RTW process through PW intervention

Theoretical methods matched with practical strategies identified for the Participatory Workplace intervention program. E = employee, OP = occupational physician, RC = RTW coordinator, S = supervisor.

Safety and equality in the PW intervention can be maintained by an independent intermediary RTW coordinator, who is able and in a key position to reach consensus between employers, OH physicians and sick-listed employees [55]. Supervisors were also identified as key intermediaries, being present every day, having the closest contact with the employees, being able to monitor a safe and appropriate modified work environment for the employees and being aware of social dynamics that may help or hinder the RTW process [55].

### Step 3 – Design a program plan

#### Focus group interviews

The focus group outcomes are summarized in four key themes: equality, safety and support in discussions about RTW; the role of the RTW coordinator; preconditions to apply the PW intervention; and expected practical barriers for the PW intervention.

#### Equality, safety and support in discussions about RTW

Discussing RTW with a supervisor was perceived as difficult by most sick-listed employees with SMDs: *'In spite of being able to get on well with my supervisor, I found talking about RTW very difficult.' *OH professionals indicated that especially with sick-listed employees with SMDs, it was difficult for supervisors to talk about RTW. The PW intervention facilitated discussion between employee and supervisor, by providing a step-by-step approach, including separate contacts between the RTW coordinator and the employee and between the RTW coordinator and the supervisor, before a discussion was held among all three. Additionally, guidance by an independent RTW coordinator acting as an intermediary ensures more equality in this discussion.

Application of the intervention for all employees is not necessary according to the employees: possibly more emancipated employees do not need the involvement of a third person. Most supervisors appreciated the well-structured protocol: *'This protocol can provide structure, and offers a framework that is useful for both employee and supervisor.' *In the opinion of the supervisors the protocol facilitates discussions about RTW in an early stage of sick leave and prevents a defensive and/or avoiding reaction from the employee. The supervisors indicated that good employee/supervisor communication was a prerequisite.

According to the employees, pressure to return does not contribute to an early RTW. Employees frequently feel guilty about sick leave. *'My supervisor frequently said how much he missed me at work. This put enormous pressure on me because I already felt guilty because I could not work.' *If a third person could help employees to them express themselves more clearly, the supervisor might understand the situation better. One employee said: *'There is a difference in authority in such a conversation with your supervisor. It would be helpful if someone monitored this process.'*

#### Role of the RTW coordinator

In the previously evaluated PW intervention the RTW coordinator did not need to be an expert on the specific complaints reported by an employee. However, in the opinion of the supervisors a RTW coordinator should be someone with experience in guidance of employees with SMDs. The employees agreed with one employee's statement: *'An intermediary must have certain expertise'*. Additionally, according to the supervisors RTW coordinators need to have knowledge about work activities in specific departments. Therefore, in this study an RTW coordinator had to be a company social worker or a labour expert. Because such professionals normally work for specific departments, all the professionals agreed that in order to ensure the independence of the intermediary position they should not be the RTW coordinator for their own department. As a consequence, it will be important that RTW coordinators explain their own specific role in this protocol.

The OH professionals indicated that the separate conversations that were held with the employee and the supervisor initially reflect both perspectives, therefore ensuring the impartial role of the RTW coordinator. Achieving equality in the conversation is another prerequisite in the expertise of the RTW coordinator, which could possibly contribute to feelings of safety for the employees. The OH professionals agreed that every solution, even if it was suboptimal according to their expertise, is appropriate if the employee and the supervisor have agreed on it. However, the suggestion that RTW coordinators could participate in the brainstorming session to derive solutions from their expertise was supported by the employees and the supervisors alike.

#### Preconditions needed for an employee before starting with the PW-intervention

The right moment to apply the PW intervention is considered to be very important. According to the OH professionals initial contact with an RTW coordinator after six weeks is too late, whereas after two weeks of sick leave employees often still lack control over their situation/complaints. *'An employee has to be able to look from some distance at his problems; this is not possible when he/she has, to a great extent, lost control.' *One employee said: *'I needed rest in the first weeks of sick leave. That is not the best time to start having these conversations.' *Some employees wanted to know more about the cause of their complaints before starting the PW intervention. This is not in line with the initial goal of the protocol: to avoid discussion about causes of sick leave and to focus on identifying barriers for RTW. Starting the intervention too late increases the risk of long-term sick leave. All three groups agreed that the timing of the application of the protocol is difficult to standardise. However, in this study there was almost unanimous agreement that the most suitable moment to start the PW intervention would be in the fourth week of sick leave. The OH professionals indicated that in some cases more consultations are needed for stress reduction and reassurance by an OP: *'Actually, reassurance and interventions for stress reduction are the first interventions that I apply, just care, take care that they get back some control.' *Regular health care needs to be maintained and the OP has a role in preventing conflicting advice about RTW. An inventory of stressors was considered to be useful to prepare conversations with the RTW coordinator. The supervisors and the employees indicated that they would appreciate checklists which support the identification of barriers for RTW.

All stakeholders agreed that it was not necessary that all complaints were alleviated before RTW. In stress-related sick leave loss of control is one of the main features and RTW can help the employee to regain control.

#### Expected practical barriers for the PW intervention

According to the stakeholders, several practical barriers, were expected to be encountered. The OH professionals expected resistance from primary care practitioners to agree to a plan for early RTW. Secondly, the employees and the OH professionals indicated the importance of appropriate and meaningful work activities. Just being present at the workplace does not encourage a full RTW. One employee said: *'Then others describe what is the best thing for you to do.' *Due to its nature, the PW intervention could prevent this situation. Thirdly, some OH professionals feared an increase in symptoms with early RTW, despite evidence for no effect on symptoms. *'The focus is on an early RTW when employees with SMDs are more vulnerable. As a result, pressure at work can possibly lead to working longer than agreed upon, which can be quite contra-productive.' *In the early stages the employees can be very vulnerable, therefore a supervisor or someone else in the department needs to check the actions formulated in the RTW plan. Fourthly, a supervisor mentioned: *'Employees frequently stick to the OP's advice about RTW.' *This advice can be a barrier for RTW, which causes problems because the supervisors are responsible for RTW. They appreciate that their role in the protocol enhances their influence on an RTW plan, even though it is time-consuming. Finally, one practical restriction could be the lack of availability of an RTW coordinator and a supervisor in planning the conversations concerning the PW intervention. At last, the employees as well as the supervisors and the OH professionals agree that implementation of the PW intervention might be feasible for employees with SMDs, taking into account the above mentioned practical barriers.

#### Processing of program plan

Table 5 presents an outline of the adapted PW intervention. The original protocol was adapted in several ways. First, the process-guiding abilities of the RTW coordinator were considered to be most important, but it was thought that some expertise in the field of SMDs was also necessary to avoid including counter-effective solutions to be included in an RTW plan. Secondly, the OP is given the opportunity to plan additional consultations before starting the PW intervention in the fourth week of sick leave. These consultations allow more attention to be paid to stress reduction techniques before starting the protocol. Thirdly, employees will be asked by their OP to fill in an inventory of stressors. It is important that stressors in the work area as well as other areas need to be specified as explicitly as possible. The employee also has to indicate the extent to which each stressor can be influenced because this will increase the employee's input in the discussions. Furthermore, the OPs are responsible for explaining the PW intervention to supervisors and asking them to participate. The OP will explain that talking about RTW in this protocol does not mean that RTW must start immediately, and that the PW intervention does not increase the complaints (based on evidence). And finally, employees can begin or continue with other treatment, if needed. The OPs explain to the employees and the supervisors that the initial RTW could be supportive, even if the complaints are still present. The OPs will send a letter about the PW intervention and a communication form to the employee's general practitioner to prevent resistance to the protocol and conflicting advice about RTW.

**Table 5:** The Participatory Workplace intervention

**Step**	**Content**	**Who is involved?**
1. Organisational preparation	Contact human resource manager or OP to provide information about who is responsible for adjustments in the workplace and what procedures should be followed	RTW coordinator
	Check that the supervisor of the employee involved has been informed about program, agrees with it and with its possible financial consequences	RTW coordinator
	Plan appointment for conversations	RTW coordinator, employee and supervisor
2. Inventory of barriers for RTW	Observation of the workplace	RTW coordinator and employee
	Interviews about tasks and barriers for RTW	RTW coordinator has separate interviews with employee and supervisor
	Prioritise barriers for RTW	Employee, supervisor and RTW coordinator
3. Thinking of, collecting solutions	Think of or collect ideas for solutions Prioritize solutions	Employee, supervisor, RTW coordinator and others
4. Preparation of the implementation	Plan the implementation of solutions.	Employee, supervisor and RTW coordinator
5. Implementing solutions	Solutions will be implemented	Depends on plan for RTW
	Visit employee to give instructions for work	RTW coordinator, employee and supervisor
6. Evaluation/control	Evaluate situation by phone: have the solutions been implemented or have improvements been made?	RTW coordinator has separate evaluations with employee and supervisor

Structure of the Participatory Workplace intervention.

The focus on barriers and solutions for RTW, rather than on causes of work disability needs to be clearly explained by OPs and RTW coordinators to avoid a conversation about the causes of sick leave. Safety and equality for employees and supervisors seem to be key factors in the RTW process, and will be monitored by the RTW coordinators. Frequently summarising the perspectives of the employee and/or the supervisor will allow them both to reformulate their perspectives. The consensus procedure will provide solutions supported by both the employee and the supervisor, and it is important that work activities are experienced as meaningful by the employee. In addition to this, the actions need to be clearly formulated in the RTW plan. Explicit arrangements about who is responsible for each solution and the period of time that it will take to implement a solution are recorded, and the anticipation of certain problems will be included in the protocol. For instance, when resistance is expected from co-workers, the supervisor can plan a meeting to explain about the PW intervention process and any possible changes. The RTW coordinator will write a report describing the barriers identified by the employee and the supervisor, selected solutions, and arrangements made in the plan for RTW. This report will be sent to the employee, the supervisor and the OP.

### Step 4 – Design an adoption and implementation plan

Adequate time, appropriate intensity of the PW intervention and sufficient resources, as well as the provision of suitable materials and training opportunities, are essential for successful implementation [20]. The protocol was also introduced at different levels in the three companies involved in the development stage, and they were all asked about their needs. The involvement of employees, supervisors and OH professionals in the planning and execution of the study will promote the transfer of the research results into daily practice [31].

A tailor-made training course was developed and planned. All the professionals involved received a syllabus, including the protocol. A separate training course was developed for the OPs and for the RTW coordinators. The training for OPs focused on: evidence that early RTW does not increase complaints, referral of employees to an RTW coordinator, the content of the protocol, and contacting supervisors to invite them to participate. The training for RTW coordinators focused on: evidence that early RTW does not increase complaints, the content of the protocol, identification of barriers and solutions for RTW, practising the protocol with anonymous cases, and reporting. Two follow-up training sessions were planned during recruitment to discuss difficulties and to practise with cases. Each RTW coordinator who guided a first case according to the protocol was contacted by the researchers to facilitate the process.

### Step 5 – Design a monitoring and evaluation plan

The effectiveness of the intervention program will be evaluated in a randomised controlled trial (RCT). Employees who have been on sick leave for 2 to 8 weeks with moderately elevated distress (measured with 3 questions of the Four-Dimensional Symptom Questionnaire distress scale [45]), will be invited to participate. This particular period of sick leave was selected because in the Netherlands the law requires that employee and employer set up plans for RTW before the 8^th ^week of sick leave. The primary outcome measure is defined as: duration of sick leave in calendar days from the first day of sick leave to full RTW, lasting at least 4 weeks without (partial or full) relapse. Psychological complaints, job content, coping, total number of days of sick leave during the follow-up period, and direct and indirect costs are secondary outcome measures. Special attention is also paid to the formulation of questions to evaluate behavioral determinants (ASE). The measurements take place at baseline and after three, six, nine and twelve months. Job stress, life-events, and problems will be considered as prognostic factors for sick leave and/or psychological complaints, and will therefore be measured at baseline. We will also conduct a process evaluation to assess satisfaction with and applicability of the protocol. The Medical Ethics Committee of the VU University Medical Center (Amsterdam, The Netherlands) has approved the study protocol. More details about the evaluation of the intervention will be presented in a separate paper.

## Discussion

The aim of this article was to describe the development, implementation and plan for the evaluation of an RTW intervention for sick-listed employees with SMDs. Although applying the IM protocol to develop this PW intervention required time and effort, it helped us to carefully consider each decision concerning the intervention in the development, implementation and evaluation phase.

### Strengths and weaknesses

IM was found to be a useful tool, although in this study the draft of the intervention protocol already existed and its effectiveness had already been demonstrated for an employee population on sick leave due to low back pain [12]. Therefore, the feasibility assessment focused on need, support from various parties and the feasibility of this intervention for employees with SMDs. It further strongly recommended systematic input from different stakeholders, such as recently sick-listed employees with SMDs, supervisors and OH professionals, thus ensuring the participation and involvement of stakeholders in all developmental stages of the program. As a result, we developed a protocol taking into account the following: a theoretical framework and different perspectives on equality, safety and support in discussions about RTW, the role of the RTW coordinator, the most suitable moment to apply the protocol, and expected barriers for the implementation of the PW intervention in individual cases. We believe that this will lead to a better compliance of OH professionals with the protocol.

In this study, only stakeholders from the participating companies were involved in the feasibility assessment and the focus group interviews. It is possible that the IM process would have led to different changes in the protocol if stakeholders from other companies were involved. It should also be noted that implementation of this intervention is likely to be more difficult in countries such as Australia or the U.S., where employer-employee relationships are more controversial.

### Comparison with other studies

Only a few publications have described the development of interventions in occupational health [56,57]. They all followed the three-phase process for conducting occupational health and safety intervention research proposed by Goldenhar and colleagues [14]: development, implementation and evaluation phases. Interviews with stakeholders, direct observation and focus groups were used to develop a tailor-made intervention in order to ensure success in the implementation and evaluation phase [56,57]. However, the main strength of IM is emphasis on a theoretical framework in combination with the involvement of stakeholders [20]. Hopefully, this approach contributes towards closing the gap between scientific evidence and daily practice in the field of occupational health.

### Recommendations

The results of this study show that IM is an appropriate tool that can be used to design interventions in OH research. Application of IM in the field of occupational health is promising, where the need for well developed tailor-made interventions is recognized by several authors [14-18]. Therefore, we would encourage designers of OH interventions and researchers investigating these interventions to go through all three phases of intervention research in collaboration with the stakeholders and to describe in more detail the development of the resulting intervention.

## Conclusion

The development of the PW intervention according to the IM protocol resulted in a structured intervention, specifically tailored to the needs of sick-listed employees with SMDs. An RCT is the next step that will be taken with great confidence in the design of the PW intervention.

The results of this RCT will be available in 2009.

## Abbreviations

RTW - Return-to-work

PW - Participatory workplace

OP - Occupational physician

OH - Occupational health

SMD- Stress-related mental disorder

IM - Intervention mapping

RCT- Randomized controlled trial

ASE- Attitude-Social influence-self-Efficacy.

## Competing interests

The author(s) declare that they have no competing interests.

## Authors' contributions

All authors have been involved in the development of the study design. SHO and JRA participated in the general coordination of the study. All authors have read and corrected draft versions of the manuscript and approved the final manuscript.

## Pre-publication history

The pre-publication history for this paper can be accessed here:


